# Harmony in Healing: Investigating Platelet-Rich Plasma Activation during Acetylsalicylic Acid Treatment

**DOI:** 10.3390/ijms252011037

**Published:** 2024-10-14

**Authors:** Małgorzata Maj, Remigiusz Tomczyk, Anna Bajek

**Affiliations:** 1 Department of Urology and Andrology, Ludwik Rydygier Collegium Medicum in Bydgoszcz, Nicolaus Copernicus University in Toruń, Karłowicza 24, 85-092 Bydgoszcz, Poland; 2 Department of Cardiology and Cardiac Surgery, 10th Military Research Hospital and Polyclinic, Powstańców Warszawy 5, 85-681 Bydgoszcz, Poland; remigiusz.tomczyk@pbs.edu.pl; 3 Department of Clinical Medicine, Bydgoszcz University of Science and Technology, Kaliskiego 7, 85-796 Bydgoszcz, Poland; 4 Department of Oncology, Ludwik Rydygier Collegium Medicum in Bydgoszcz, Nicolaus Copernicus University in Toruń, Łukasiewicza 1, 85-821 Bydgoszcz, Poland; a.bajek@cm.umk.pl

**Keywords:** antiplatelet drugs, collagen, inflammatory cytokines, platelet-rich plasma, regenerative therapies, thrombin

## Abstract

Platelet-rich plasma (PRP) therapy holds promise for treating various clinical conditions. The activation process is crucial in releasing growth factors and cytokines from platelets, enhancing the therapeutic properties of PRP. Standard activation methods involve autologous thrombin or collagen, with variations in efficacy and growth factor release. This study explores the impact of acetylsalicylic acid (ASA), a commonly used antiplatelet drug, on PRP activation. The results indicate that non-activated PRP extracted from the whole blood of ASA-treated patients exhibits increased inflammatory cytokine concentrations, notably TNFa. After activation with autologous thrombin/CaCl_2_ or collagen IV, the measured fluorescence intensities suggest varying release patterns between treated and non-treated groups. Understanding the influence of ASA on platelet activation holds implications for personalized medicine and optimizing outcomes for individual patients undergoing PRP therapy. This research sheds light on the potential challenges associated with using antiplatelet drugs, emphasizing the need for careful consideration in tailoring PRP-based regenerative therapies.

## 1. Introduction

Platelet-rich plasma (PRP) therapy involves the extraction and concentration of a patient’s blood to create a solution with a higher concentration of platelets than in the whole blood. It is generally accepted that a platelet concentration in PRP should exceed 1x10^6^/μL or increase three- to five-fold from baseline [1,2]. PRP was successfully used to treat various clinical conditions, including musculoskeletal diseases, chronic ulcers, and hard-to-heal wounds [2,3,4]. It is also used in dermatology and esthetic medicine to stimulate fibroblast activity and the production of collagen and elastin [5,6]. Platelets play a crucial role during the body’s natural healing process, as they contain various growth factors and proteins that can promote tissue repair and regeneration. The activation process is essential as it triggers the release of growth factors and bioactive proteins from platelets, enhancing the therapeutic properties of PRP [7].

PRP may be activated using autologous thrombin (alone or combined with calcium chloride) isolated from platelet-poor plasma extracted along with PRP. Thrombin converts soluble fibrinogen to fibrin and, as a result, creates a dense network of fibrin fibers [8]. However, relying on in situ collagen activation is common in clinical practice. As activation with collagen does not result in clot formation, platelets and released growth factors may migrate away from the activation site. On the other hand, collagen activation results in lower growth factor release, so it may be used in clinical applications where a slow and gradual release seems beneficial [9]. As the field evolves, researchers explore novel, experimental methods of PRP activation, e.g., combining extracellular calcium and electric pulses [4,10].

PRP activation methods can influence the effectiveness of PRP therapy, and the choice of activation method may be considered in a personalized medicine approach to optimize outcomes for individual patients. The use of antiplatelet medications may reduce the effectiveness of PRP therapy, as the injected platelets may be less active in promoting healing due to the ongoing inhibition by the drugs. It was shown that the antiplatelet effects of acetylsalicylic acid might last for 96 h after its administration [11,12]. Therefore, the reduced activation of platelets isolated from patients’ blood using acetylsalicylic acid or other antiplatelet drugs may influence the efficacy of PRP-based regenerative therapies. For that reason, this preliminary study aimed to analyze the influence of aspirin, one of the most commonly used drugs in the world, on platelet activation.

## 2. Results

### 2.1. Platelet Concentration in the Whole Blood

The mean number of platelets in the blood of patients treated with acetylsalicylic acid was 269.33 10^3^/μL ± 29.48 (Table 1). In the blood of patients without antiplatelet medication, the mean number of platelets was 217.67 10^3^/μL ± 48.13. The difference between groups was not statistically significant. All results were in the normal range (132 − 370 10^3^/μL).

### 2.2. Clot Formation

**Figure 1:**
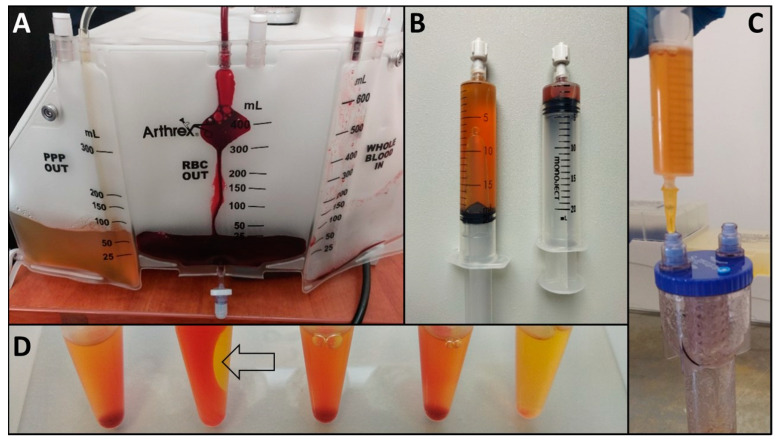
Platelet-rich plasma collection and activation. PRP was isolated using a whole blood separator (**A**). PPP (syringe on the left) was collected each time along with the PRP (**B**) and was used for subsequent thrombin isolation (**C**). Activation with thrombin resulted in visible clot formation, marked with a black arrow (**D**). Samples after 60 min activation, from the left: non-activated PRP, PRP activated with thrombin, PRP activated with collagen IV, PRP activated with collagen I, and PPP. PRP—platelet-rich plasma; PPP—platelet-poor plasma.

Thrombin isolated from PPP, collected from each patient along with PRP (Figure 1A–C), induced clot formation (Figure 1D, black arrow). In turn, both collagens failed to induce platelet gel formation. No visible clot could be observed even after 60 min incubation.

### 2.3. Inflammatory Cytokines in Non-Activated PRP

**Figure 2:**
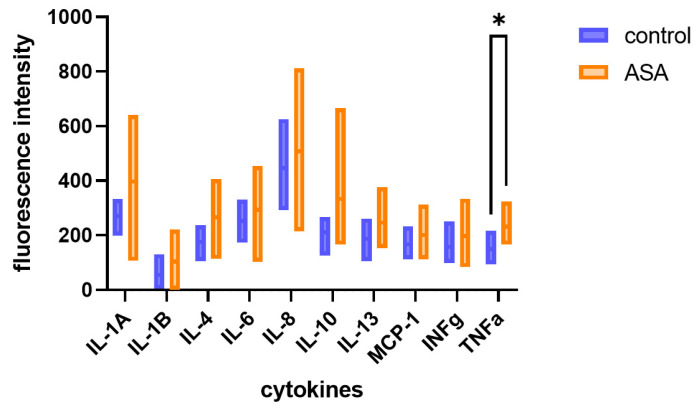
The comparison between cytokine concentration in non-activated platelet-rich plasma obtained from patients treated with acetylsalicylic acid (ASA) compared to the control group. After activation with autologous thrombin/CaCl_2,_ the fold increase in fluorescence intensity in the ASA group in comparison to the control group was 1.16 for IL-1A, 1.10 for IL-8, 1.16 for IL-10, 1.06 for MCP-1, 1.11 for INFg, and 1.32 for TNFa (Figure 3). For IL-1B, IL-4, and IL-6, activation with thrombin/CaCl2 resulted in lower fluorescence intensity in the ASA group than in the control. Activation with collagen I increased only the release of MCP-1 (1.15-fold) and TNFa (1.26-fold). In turn, activation with collagen IV resulted in a fold change of 1.61, 1.38, 1.10, 1.22, 1.23, 1.48, and 1.10 for IL-1B, IL-4, IL-6, IL-8, IL-10, IFNg, and TNFa, respectively. *, *p*-value < 0.05 was considered statistically significant.

In patients treated with acetylsalicylic acid, increased inflammatory cytokine concentration could be noted in non-activated PRP (Figure 2). The fold increase in fluorescence intensity that corresponds to concentration in the ASA group in comparison to the control group was 1.47 for IL-1A, 1.92 for IL-1B, 1.51 for IL-4, 1.16 for IL-6, 1.14 for IL-8, 1.58 for IL-10, 1.32 for IL-13, 1.20 for MCP-1, 1.25 for INFg, and 1.54 for TNFa. The results were significant only for TNFa. Nevertheless, median values for all tested cytokines, both pro-inflammatory and anti-inflammatory, were higher in the treated group than in the control group.

## 3. Discussion

Platelet-rich plasma has demonstrated its efficacy in enhancing wound healing across various conditions, including chronic diabetic ulcers, pressure ulcers, and acute traumatic wounds [13,14]. Additionally, PRP has proven to be a safe and effective treatment for different types of atrophic scars. According to a meta-analysis, patients treated with PRP exhibited an overall response rate of 23%, comparable to those observed with laser or micro-needling (22% and 23%, respectively). When PRP was used alone, moderate improvement was the most frequently observed degree of response. However, when combined with laser or micro-needling, many patients experienced marked results or excellent outcomes [15]. Controlled studies have also indicated that incorporating two-to-four sessions of PRP with traditional therapies and procedures can effectively minimize acne scarring and facial burns, improve esthetic results, and reduce recovery time [16].

PRP activation is required to stimulate tissue regeneration and repair. One of the most common methods involves using autologous thrombin and CaCl_2_. As a result of platelet degranulation, more than 300 proteins are released, including growth factors and cytokines, that stimulate the proliferation, migration, and differentiation of cells within the tissue microenvironment [17]. It was previously shown that the activation method influences the physical form of PRP. Thrombin, CaCl_2_, thrombin combined with CaCl_2_, and collagen I induced different platelet aggregation. PRP activated with thrombin alone or combined with CaCl_2_ formed stable clots after 15 min of incubation (stable up to 24 h). In turn, no clots were noted in samples activated with collagen [9]. We observed similar differences in platelet aggregation (Figure 1D). Stable clots were formed after PRP activation with thrombin combined with CaCl_2_. Clot formation could not be noted after collagen I or IV activation. The same effects were observed in the ASA-treated and non-treated groups (Table 1).

PRP-based therapies are hindered by variations in PRP formulations, inconsistencies in nomenclature, and a need for standardized, evidence-based guidelines. Amable et al. addressed these challenges by establishing an optimized method for PRP preparation. Their method demonstrated consistency across different blood donors, recovering 46.9% to 69.5% of total initial platelets and resulting in a 5.4-fold to 7.3-fold increase in platelet concentration. The researchers also quantified growth factors, cytokines, and chemokines released by the platelets after activation with CaCl_2_ and CaCl_2_ combined with thrombin. High PDGF, EGF, and TGF concentrations and anti-inflammatory and pro-inflammatory cytokines, including IL-4, IL-8, IL-13, IL-17, TNFa, and IFNg, were observed [18]. In our study, following activation with autologous thrombin combined with CaCl_2_, the ASA group exhibited an increase in fluorescence intensity compared to the control group for IL-1A (1.16-fold), IL-8 (1.10-fold), IL-10 (1.16-fold), and TNFa (1.32-fold). In contrast, for IL-1B, IL-4, and IL-6, activation resulted in lower fluorescence intensity in the ASA group than in the control. Activation with collagen I decreased fluorescence intensity for all tested pro-inflammatory cytokines except TNFa (1.26-fold). Collagen IV increased the concentration of all tested cytokines except IL-13, notably INFg (1.48). The initial elevated TNFa concentration in non-activated PRP likely influenced the cytokine profile after activation, particularly with thrombin/CaCl_2_ and collagen IV, where TNFa levels stayed high. This highlights the importance of considering pre-activation cytokine levels and the choice of activation method when using non-activated PRP in future studies, as elevated inflammatory cytokine levels, especially TNFa and INFg, could significantly influence therapeutic outcomes, particularly in cases where inflammation modulation is crucial.

Pochini et al. used samples from six individuals to obtain PRP using the open method and commercial columns, revealing that the concentration of growth factors and cytokines varied based on the centrifugation technique. The open system yielded elevated levels of IL-6, while concentrations of other analyzed cytokines and chemokines were comparable between systems [19]. In a separate study, researchers comparing the content, repeatability, and correlations of biologically active components in PRP obtained through different methods observed significantly higher concentrations of platelets, WBC, and RBC in PRP obtained with Mini GPS III. Notably, the highest concentrations of EGF, VEGF, HGF, PDGF-AA, and PDGF-BB were found in PRP samples obtained with Mini GPS III, whereas the lowest levels were observed in samples obtained with Arthrex ACP [20]. Oh et al. evaluated PRP’s cellular composition and cytokine-release kinetics according to differences in the preparation protocols. The double-spin method led to a higher platelet concentration than the single-spin method. However, the cytokine content did not correlate proportionally with the cellular composition of the PRPs [21]. Another factor influencing PRP activity and cytokine release may be storage time and temperature, as discovered by Kim et al. [22]

Niemann et al. used three PRP preparations to concentrate whole blood samples from twelve healthy donors. They found that each participant’s immune profile was maintained in the final PRP products, with significant variations in cytokine compositions between products. Additionally, pro-inflammatory cytokines negatively impacted the phenotype and function of chondrocytes [23]. Mochizuki et al. discovered that PDGF and TGFβ1 levels in leukocyte-rich PRP (LR-PRP) were significantly lower in female athletes than in ordinary healthy adults. In comparison, IL-1β and IL-1RA levels were higher, suggesting potentially more favorable clinical outcomes [24]. Wang et al. summarized the feasibility and mechanisms of PRP-based growth factors in osteoarthritis. They concluded that not all cytokines released by PRP are beneficial, although the therapeutic action of PRP has a valuable potential to improve [25]. All these results indicate the need for careful characterization of PRP products to treat various pathologies under specific conditions.

The influence of medications, such as non-steroidal anti-inflammatory drugs, on platelet secretome release is a notable factor. Jayaram et al. conducted a study with samples from twelve healthy men, employing a double-spin protocol to prepare RL-PRP. This process was repeated after 14 days with an 81 mg daily dose of oral ASA. Interestingly, a 14-day regimen of daily ASA demonstrated no impact on the quantified number of platelets and leukocytes in whole blood and LR-PRP. However, the everyday use of low-dose ASA did reduce the expression of VEGF, PDGF-AB, and TGF-B in freshly isolated human LR-PRP when activated with arachidonic acid. ASA exhibited no effect on thrombin-mediated VEGF and TGF-B release from LR-PRP but partially inhibited PDGF-AB release [26]. A systematic review by Frey et al. suggested that antiplatelet medications could decrease the growth factor release profile depending on cyclooxygenase 1 and cyclooxygenase 2. Eight out of fifteen studies in the review reported a decrease in growth factor release [27].

Previous studies have shown the gradual recovery of platelet activity within 72 h after antiplatelet therapy withdrawal, with complete normalization after 96 h [11,12,28]. For that reason, Ludwik et al. explored a 3-day washout to assess its effectiveness in mitigating cyclooxygenase-2 inhibitor-related impairments on platelet function in a canine model. Interestingly, the inhibitor did not impair platelet activation, growth factor release, or TXB2 production in canine PRP when human thrombin was used as an activator [29]. Further studies using human samples are required to determine the time to minimize the influence of aspirin and other antiplatelet drugs on platelet activity using standardized PRP formulations.

## 4. Materials and Methods

### 4.1. PRP Collection

Platelet-rich plasma (PRP), platelet-poor plasma (PPP), and autologous thrombin were obtained from six patients at the Cardiology Clinic of Antoni Jurasz University Hospital No. 1 in Bydgoszcz, Poland. These patients, who were receiving acetylsalicylic acid (ASA) as part of their routine treatment, had an additional 40 mL of blood collected during their regular tests specifically for this study. PRP and PPP were prepared using the Arthrex Angel System (Arthrex, Munich, Germany), which separates whole blood into its components. The processing protocol was set for a hematocrit of 4%. PRP was extracted from the buffy coat, while PPP was collected separately, both into sterile syringes.

The study received approval from the Bioethics Committee of Nicolaus Copernicus University in Torun, Ludwik Rydygier Collegium Medicum in Bydgoszcz (KB 344/2020). All procedures were conducted strictly with applicable guidelines and regulations, and informed consent was obtained from all participants.

### 4.2. Thrombin Isolation

Thrombin was isolated each time from PPP collected along with PRP using a Thrombinator System (Arthrex, Munich, Germany). In total, 0.1 mL of CaCl_2_ and 4 mL of autologous blood fraction were added to the system. After 10 min incubation, the clot was broken by vigorous shaking. Then, 0.2 mL of CaCl_2_ and 8 mL of autologous blood fraction were added. After 1 min incubation, the clot was broken, and the filter was placed on the withdraw port of the device. Filtered serum was ready to use for platelet activation.

### 4.3. PRP Activation

The activation of PRP was performed by adding autologous thrombin and CaCl_2_ (described above), collagen type I, or collagen type IV (final concentration 4 μg) (Thermo Fisher Scientific, Waltham, MA, USA). PRP without activation was used as a control. Blood derivatives were incubated for 60 min. Then, the samples were centrifuged at 2800× *g* for 15 min at RT, and the supernatants were collected and stored at −80 °C until use.

### 4.4. Cytokine Release

PRP and PPP were evaluated for the release of inflammatory cytokines (IL-1A, IL-1B, IL-4, IL-6, IL-8, IL-10, IL-13, MCP-1, IFNg, and TNFa) using Quantibody Human Inflammation Array 1 (RayBiotech, Peachtree, Corners, GA, USA). Slides were blocked with 100 μL of Sample Diluent for 30 min. Then, the samples were added, and the slides were incubated overnight at 4 °C. The slides were washed with Wash Buffer I and II and incubated with the biotinylated antibody for 2 h at RT. Then, the slides were washed with Wash Buffer I and II and incubated with Cy3 equivalent dye-conjugated streptavidin. After washing, the slides were dried with a compressed N2 stream and sent to the manufacturer for visualization.

### 4.5. Statistical Analysis

The results of at least three independent measurements were presented as means ± standard deviation (SD). The Shapiro–Wilk test was employed to assess the normality of data sets, followed by Student’s t-test, which was used to compare the means between the two groups. A *p*-value < 0.05 was considered statistically significant. All analyses were performed using STATISTICA 13.1 (StatSoft, Cracow, Poland).

## 5. Conclusions

Challenges in platelet-rich plasma therapy arise from individual variations and diverse preparation methods. Variability in patient responses and outcomes is evident due to differences in immune profiles, cellular compositions, and cytokine concentrations among individuals. Additionally, PRP formulations obtained through various techniques exhibit fluctuations in growth factor levels, impacting therapeutic efficacy. The standardization of preparation methods, guided by evidence-based protocols, is crucial to ensure consistency and optimize the therapeutic potential of PRP. Addressing these challenges will enhance the reliability and effectiveness of PRP therapies, providing more predictable outcomes for diverse patient populations, especially those treated with antiplatelet drugs.

## Figures and Tables

**Figure 3:**
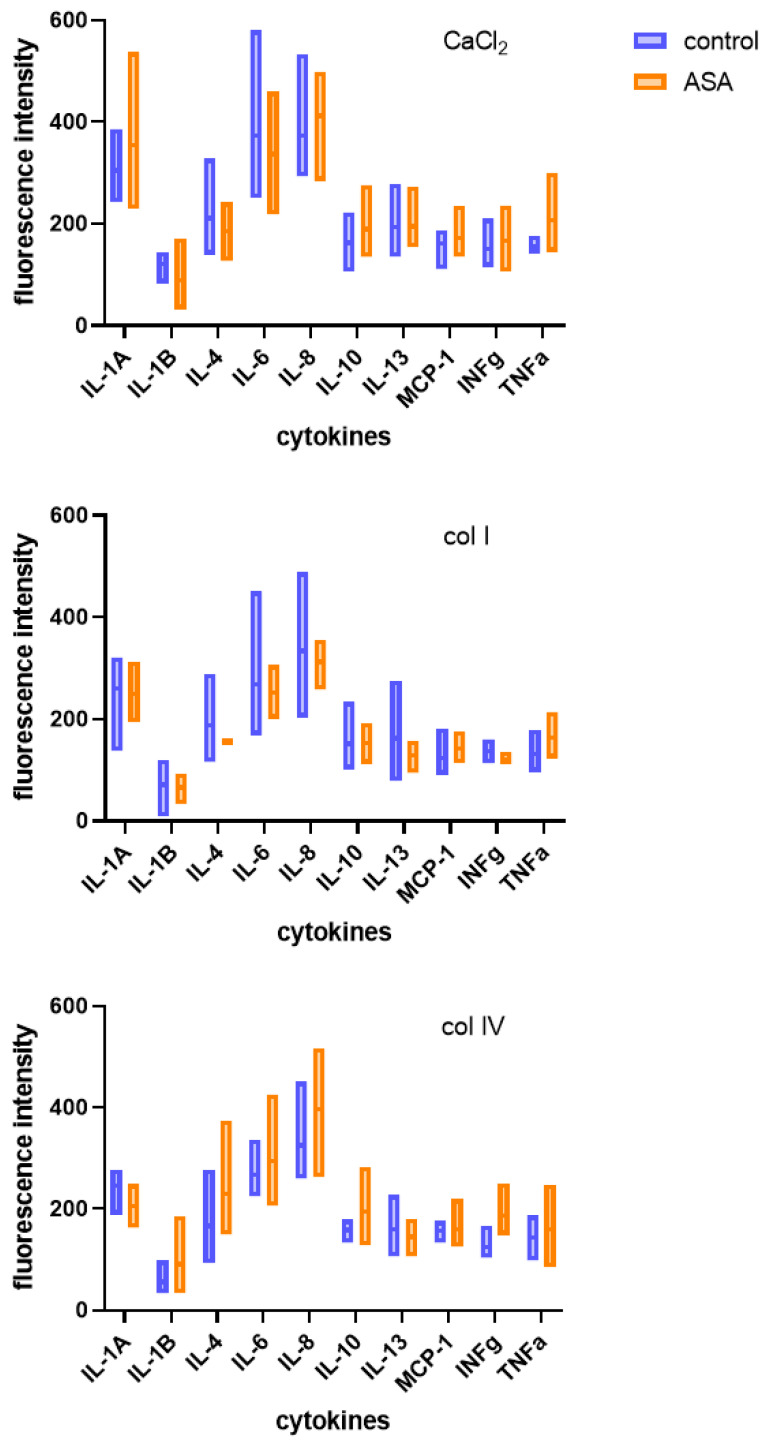
The comparison between inflammatory cytokine concentration in platelet-rich plasma activated with calcium chloride (CaCl_2_), collagen I (col I), and collagen IV (col IV) obtained from patients treated with acetylsalicylic acid (ASA) compared to the control group.

**Table 1:** Complete blood count of the patients in the study. Data are presented as mean ± SD (in brackets).

	WBCs [10^3^/μL]	RBCs [10^6^/μL]	HGB [g/dL]	HCT [%]	PLT [10^3^/μL]
Control	6.55 (1.67)	4.57 (0.24)	13.97 (0.40)	40.67 (0.23)	217.67 (48.13)
ASA	8.10 (1.56)	4.91 (0.18)	14.70 (0.79)	43.43 (1.65)	269.33 (29.48)

There were no statistically significant differences between the analyzed parameters. ASA—acetylsalicylic acid; HCT—hematocrit; HGB—hemoglobin; PLT—platelets; RBCs—red blood cells; WBCs—white blood cells.

## Data Availability

The data presented in this study are available in the article.
